# Community Social Capital and Depressive Symptoms Among Older People in Japan: A Multilevel Longitudinal Study

**DOI:** 10.2188/jea.JE20180078

**Published:** 2019-10-05

**Authors:** Miwa Yamaguchi, Yosuke Inoue, Tomohiro Shinozaki, Masashige Saito, Daisuke Takagi, Katsunori Kondo, Naoki Kondo

**Affiliations:** 1 Department of Nutrition and Metabolism, National Institutes of Health and Nutrition, National Institutes of Biomedical Innovation, Health and Nutrition, Tokyo, Japan; 2 Carolina Population Center, The University of North Carolina at Chapel Hill, Chapel Hill, NC, USA; 3 Department of Biostatistics, School of Public Health, the University of Tokyo, Tokyo, Japan; 4 Department of Social Welfare, Nihon Fukushi University, Aichi, Japan; 5 Center for Well-being and Society, Nihon Fukushi University, Aichi, Japan; 6 Department of Health and Social Behavior, School of Public Health, the University of Tokyo, Tokyo, Japan; 7 Department of Health Education and Health Sociology, School of Public Health, the University of Tokyo, Tokyo, Japan; 8 Center for Preventive Medical Sciences, Chiba University, Chiba, Japan; 9 Department of Gerontological Evaluation, Center for Gerontology and Social Science, National Center for Geriatrics and Gerontology, Aichi, Japan

**Keywords:** civic participation, late-life depression, multilevel analysis, reciprocity, social cohesion

## Abstract

**Background:**

This study aimed to examine the contextual effects of community-level social capital on the onset of depressive symptoms using a longitudinal study design.

**Methods:**

We used questionnaire data from the 2010 and 2013 waves of the Japan Gerontological Evaluation Study that included 14,465 men and 14,600 women aged over 65 years from 295 communities. We also used data of a three-wave panel (2006–2010–2013) to test the robustness of the findings (*n* = 7,424). Using sex-stratified multilevel logistic regression, we investigated the lagged associations between three scales of baseline community social capital and the development of depressive symptoms.

**Results:**

Community civic participation was inversely associated with the onset of depressive symptoms (men: adjusted odds ratio [AOR] 0.93; 95% confidence interval [CI], 0.88–0.99 and women: AOR 0.94; 95% CI, 0.88–0.997 per 1 standard deviation unit change in the score), while no such association was found in relation to the other two scales on social cohesion and reciprocity. This association was attenuated by the adjustment of individual responses to the civic participation component. Individual-level scores corresponding to all three community social capital components were significantly associated with lower risks for depressive symptoms. The results using the three-wave data set showed statistically less clear but similar associations.

**Conclusions:**

Promoting environment and services enhancing to community group participation might help mitigate the impact of late-life depression in an aging society.

## INTRODUCTION

Social capital, defined as the resources that are accessed by individuals as a result of their membership of a network or group,^1^ has been suggested to be associated with health outcomes, while some studies also revealed inverse associations.^2^^,^^3^ There are several categorizations of social capital in the existing literature, reflecting the different foci of its forms and dimensions, as follows: (1) level of measurement (ie, individual-level vs community-level social capital); (2) subjective or objective (ie, cognitive vs structural social capital); and (3) types of typical relationships among members (ie, bonding vs bridging social capital).^1^

Mental disorders, including depression, have been linked with social capital.^4^^–^^7^ For example, systematic reviews by De Silva et al and Ehsan and De Silva indicated strong evidence of an inverse association between individual cognitive social capital and common mental disorders (CMD),^5^^,^^6^ while the association with individual structural social capital was reported to be less consistent in previous literature. At a community level, Ehsan and De Silva concluded that higher cognitive social capital was associated with lower risk of CMD in seven cross-sectional studies, but that structural social capital was not associated with CMD.^6^

Several pathways have been proposed to explain the mechanism linking community social capital and depressive symptoms, including collective efficacy, informal social control, and social influence.^8^^,^^9^ For example, communities rich in social capital can influence political decisions to make healthcare services readily available in their communities (ie, collective efficacy).^8^^,^^9^ Health-related information or positive feelings are known to be transmitted more rapidly in communities with high social capital (ie, social influence).^9^ In addition, living in communities with higher neighborhood safety as a result of informal social control may reduce psychological stress.^9^

These potential contextual effects may directly affect health regardless of individual behaviors, but they may also enhance individual-level pathways. For instance, sense of belonging may improve by participating in community organizations (related to community civic participation) and living in communities whose residents are trustworthy (related to community social cohesion).^10^^–^^12^ This sense of belonging may improve people’s ability to cope with stressors, and thus may have protective effects against the onset or progression of depressive symptoms. Social support also helps people cope with stressors (related to community reciprocity).^13^ Increased physical activity owing to civic participation has also been shown have such protective effects.^14^^,^^15^

The present study was designed to extend the findings of previous studies on the association between social capital and depression. First, despite the existence of some cross-sectional studies,^16^^,^^17^ few studies have investigated the association between social capital and mental illnesses using a longitudinal design while accounting for both community-level social capital variables and individual-level responses.^18^ It is essential to include both community- and individual-level variables in a model to evaluate the contextual effects of community social capital (ie, to study the effects of residing in a community on having a certain level of social capital regardless of the level of social capital an individual holds).^11^^,^^19^ Second, we examined the effect of community-level social capital on depressive symptoms of the older population in Japan, a country experiencing rapid population aging. Given that depression is a mental disorder common in older people^20^ and that late-life depression is linked with a variety of negative health outcomes,^21^ it has become important to identify factors that help mitigate the impact of depression in aging societies. Third, we used study-specific social capital measures that have been validated in relation to depressive symptoms and self-rated health in the current study population of older people living in Japan.^22^ While these scales were originally developed to measure community-level social capital, we also used individual responses as indicators of individual-level social capital in this study, as they were deemed to reflect individual-level social capital in terms of neighborhood organization participation, individual perception of community cohesion, and availability of mutual support.^23^^,^^24^

Thus, the present study aimed to investigate the contextual effects of community-level social capital on the onset of depression using longitudinal panel data from the Japan Gerontological Evaluation Study (JAGES).

## METHODS

### Study participants

Data was obtained from the JAGES, which is a prospective cohort study that examined the influence of social determinants of health among older people in Japan.^25^^,^^26^ For the present study, we used longitudinal data from the 2010 and 2013 waves; in the 2010 wave, data were collected from 112,123 residents (response rate: 66.3%) aged 65 years or older who did not receive long-term care and resided in 533 school districts (ie, elementary school or junior high school, depending on municipalities) in 31 municipalities. The follow-up survey was conducted among the residents who participated in the 2010 wave, and 62,438 participants responded to the 2013 wave. We excluded 555 participants with self-reported difficulties in activities of daily living (ie, walking, toileting, and bathing) at the 2010 wave. After excluding those who lived in school districts in which fewer than 50 residents responded (*n* = 7,025),^22^ those with missing information on self-reported depressive symptoms (*n* = 16,350), and those who reported to have depression/depressive symptoms in the 2010 wave (ie, the Geriatric Depression Scale (GDS) score ≥5 [described later] or previous diagnosis of depression) (*n* = 9,443), a total of 29,065 subjects (14,465 men and 14,600 women) nested within 295 school districts were included in the subsequent analyses.

We also created an alternative three-wave dataset using the 2006, 2010, and 2013 waves. As the 2006 wave was conducted only in 25 districts in Chita Peninsula, Aichi Prefecture, the sample size was 7,424 (3,779 men and 3,645 women). We excluded those who had depression/depressive symptoms at an earlier wave to investigate the association at the subsequent waves.

The JAGES protocol and informed consent procedure were approved by the Ethics Committee for Research of Human Subjects at Nihon Fukushi University (no. 10-05 and no. 13-14) and the Ethics Committee for Medical Research at the University of Tokyo (no. 10555).

### Social capital

We used the study-specific health-related community social capital indicators validated in the JAGES population.^22^ The scale comprised three dimensions: civic participation (five questions), social cohesion (three questions), and reciprocity (three questions). Civic participation refers to the level of residents’ participation in community organizations and activities.^27^^,^^28^ Social cohesion pertains to the cognitive aspects of interpersonal trust, reciprocity, and attachment to the community.^28^^,^^29^ It was assessed using the questions, “Generally speaking, would you say that most people can be trusted?”, “Do you think that it is important to be helpful to other people in your local area?”, and “Do you have an attachment to your local area?”. Responses options were presented as a Likert scale (1 = strongly agree/yes, 2 = moderately agree/yes, 3 = neither, 4 = disagree/no). Respondents who chose “strongly or moderately agree/yes” were coded as 1, and were otherwise coded as 0. These scores were then summed to compute a composite score ranging from 0–3. In our three-wave analysis, as information on attachment to the community was not available in the 2006 wave, we calculated the score of social cohesion based on scores on community trust and community contribution (score range: 0–2). Reciprocity represents the community characteristics of exchanging support,^3^ and the following three questions were used in the scale: “Do you have someone who listens to your concerns and complaints?”, “Do you listen to someone’s concerns and complaints?”, and “Do you have someone who looks after you when you are sick and confined to bed for a few days?”. Respondents who chose “strongly or moderately agree/yes” were coded as 1 and were otherwise coded as 0 to compute a composite score ranging from 0–3.

Following Saito et al^22^ and other previous literature,^11^ we aggregated individual-level scores on the three types of social capital to obtain community-level scores by school district (*n* = 295). Specifically, the average scores of individual-level responses in relation to civic participation, social cohesion, and reciprocity were calculated for each school district. School district is the smallest area unit available in the JAGES. Historically, school districts are likely to represent the unit of former “villages” before repeated municipality mergers took place in the last few decades in Japan.^30^ For comparison between the waves, we standardized the community-level and individual-level scores to a mean of 0 and standard deviation (SD) of 1.

Since the 2006 and 2010 waves lacked data on two questions in relation to for civic participation (participation in “study or cultural groups” and “activities for teaching specific skills”), the present study used three out of five of the original questions. Consequently, civic participation in the 2006 and 2010 waves was measured by asking if the respondents participated in volunteer groups, hobby groups, and sports groups. Following a previous study,^22^ those who participated in these organizations once a month or more were categorized as having a high level of participation. This civic participation score ranged from 0 to 3. By conducting confirmatory factor analyses using data of the 2013 wave, limited to the questions we used in the present study, we investigated that our social capital measures, lacking two questions in our sample for the two-wave analysis, and an additional question for the three-wave analysis, had factor components similar to those of their original versions provided by Saito et al.^22^

### Depressive symptoms

To evaluate depressive symptoms, we used the short form of the GDS, comprising 15 questions (GDS-15; score range: 0–15; Cronbach’s alpha = 0.80; Area under the ROC curve = 0.98).^31^^–^^33^ Participants were judged to have depressive symptoms when they had a score of 5 points or above^30^^,^^31^ or when they reported a previous diagnosis of depression. The outcome was defined as the onset of depressive symptom between the baseline (2010) wave and the follow-up (2013) wave.

### Covariates

As potential confounders, we adjusted for the following covariates in our analysis, measured at the 2010 wave: age (65–69, 70–74, 75–79, and ≥80 years), family structure (living alone, living with family, and others), marital status (married, divorced, widowed, and never married), equivalent household income (ie, total household income divided by the square root of the number of household members; <2.00, 2.00–3.99, and ≥4.00 million yen), current employment (working, not working, and never working), educational attainment (years of schooling: ≤9, 10–12, and ≥13 years), and comorbidity (heart disease, stroke, diabetes mellitus, and cancer). We used these four diseases following a previous study on social capital and depression.^34^ If participants had missing information for any of the covariates, they were assigned to a “missing” category and included in the analysis.

### Statistical analysis

To investigate the association between community-level social capital and the onset of depressive symptoms, we used a sex-stratified multilevel logistic regression. After confirming the presence of suggestive interaction between sex and social capital (eTable 1), we stratified the data by sex; this process was also in line with previous studies in Japan that reported gender differences in the association between community-level social capital and depressive symptoms.^35^^–^^37^ We modeled the onset of depressive symptoms (GDS ≥5) between 2010 and 2013, incorporating one of the three community-level social capital components (civic participation, social cohesion, or reciprocity) at baseline. The models were as follows:

$$\begin{array}{l} \text{logitPr}\;(\text{Depressive}\;{\text{symptoms}}_{ij,\text{2013}})\\ \;=\mu +b_{i}+\alpha \cdot {\text{ISC}}_{ij,\text{2010}}+\beta \cdot {\text{CSC}}_{j,\text{2010}}+\gamma \cdot C_{ij,\text{2010}} \end{array}$$

where probability individual *i* in school district *j* having depressive symptoms at 2013 was modeled by individual responses to social capital components (ISC) and community-level social capital scores (CSC) at baseline (2010), controlling for their covariates (*C*) and random intercepts for school districts (*b_i_*).

For the three-wave analysis, we included those who participated in the study at least twice (ie, those who participated in 2006 and 2010; 2010 and 2013; 2006 and 2013; and 2006, 2010 and 2013). The models were modified as follows:

$$\begin{array}{l} \text{logitPr}\;(\text{Depressive}\;{\text{symptoms}}_{ij,\text{2010}})\\ \;=\mu_{2010}+b_{i}+\alpha \cdot {\text{ISC}}_{ij,\text{2006}}\text{+}\beta \cdot {\text{CSC}}_{j\text{2006}}\text{+}\gamma \cdot {\text{C}}_{ij,\text{2006}} \end{array}$$

$$\begin{array}{l} \text{logitPr}\;({\text{Depressive symptoms}}_{ij,\text{2013}}|{\text{No Depressive symptoms}}_{ij,\text{2010}})\\ \;=\mu_{2013}+b_{i}+\alpha \cdot {\text{ISC}}_{ij,\text{2010}}\text{+}\beta \cdot {\text{CSC}}_{j,\text{2010}}\text{+}\gamma \cdot {\text{C}}_{ij,\text{2010}} \end{array}$$

That is, we modeled the discrete hazards of the onset of depressive symptoms by social capital measured in an earlier wave with time-invariant effects and time-specific intercepts. For those who participated at the 2006 and 2013 waves, the predictor variables were substituted by those measured at 2006 (ie, ISC*_ij_*_,2006_, CSC*_j_*_,2006_, and *C_ij_*_,2006_), assuming that their variables were stable over the 2006–2010 period. We first tested a null model to evaluate the community level variability in the onset of depressive symptoms. Next, in model 1, we examined the association between community-level social capital and the onset of depressive symptoms separately, while adjusting for possible covariates at baseline. Then, in model 2, we modeled the community-level social capital scores by further adjusting for ISC scores. We provide the adjusted odds ratios (AORs) and their 95% confidence intervals (CIs). Based on the random-effects variance, we calculated the median odds ratio (MOR) to examine the unexplained heterogeneity between communities. If there was a strong difference between school districts, the MOR would be further away from 1.^38^ All statistical analyses were performed using Stata 14.0 (StataCorp LP, College Station, TX, USA). The level of statistical significance was set at *P* < 0.05 (two-tailed).

## RESULTS

The results of the confirmatory factor analysis for the questions used to evaluate social capital in the present study showed that they had the same components as the original measure (eTable 2 and eTable 3). Table 1 shows baseline distributions of the civic participation, social cohesion, and reciprocity scores at individual and community levels. In general, the distribution of the individual-level scores of civic participation was skewed towards low scores, whereas the individual-level scores of social cohesion and reciprocity were skewed towards high scores.

**Table 1.** Individual- and community-level distributions of the civic participation, social cohesion, and reciprocity scores at baseline (2010)

	Individual level^a^						Community level^c^	
	
			0 (no score)	1	2	3		
	*n*	Median^b^	*n* (%)	*n* (%)	*n* (%)	*n* (%)	*n*	Median (IQR)
**Men**								
Civic participation	12,051	−0.88	6,301 (52.3)	3,007 (25.0)	2,164 (18.0)	579 (4.8)	14,465	−0.15 (−0.60, 0.34)
Social cohesion	13,828	0.73	834 (6.0)	1,924 (13.9)	2,852 (20.6)	8,218 (59.4)	14,465	0.27 (−0.15, 0.68)
Reciprocity	13,717	0.25	106 (0.8)	359 (2.6)	667 (4.9)	12,585 (91.8)	14,465	0.31 (−0.03, 0.63)
**Women**								
Civic participation	10,871	0.20	4,900 (45.1)	2,968 (27.3)	2,379 (21.8)	624 (5.7)	14,600	−0.20 (0.62, 0.26)
Social cohesion	13,873	0.73	1,029 (7.4)	2,134 (15.4)	2,791 (20.1)	7,919 (57.0)	14,600	0.27 (−0.15, 0.68)
Reciprocity	13,720	0.25	46 (0.3)	136 (1.0)	595 (4.3)	12,943 (94.3)	14,600	0.32 (−0.03, 0.63)

IQR, interquartile range.

^a^The social capital score at the individual level was the total number of “strongly or moderately agree/yes” responses to civic participation, social cohesion, and reciprocity, respectively (eg, a 0 score indicated that none of items apply, while a 3 score indicated that all the items apply).

^b^Median of the standardized social capital score.

^c^Standardized percentages of average number of “strongly or moderately agree/yes” responses in a school district.

The mean age of the participants was 72.3 years (SD, 5.4) for men and 72.4 years (SD, 5.4) for women. Most of the participants lived with their family and were likely to be married, retired, and have a middle socio-economic status according to the household income and years of schooling (Table 2). Approximately half of the participants had no comorbidity related to depressive symptoms.

**Table 2.** Baseline characteristics of the study participants in the Japan Gerontological Evaluation Study (2010)

	Men	Women
Age, years		
65–69	5,526 (38.2)	5,419 (37.2)
70–74	4,456 (30.8)	4,602 (31.5)
75–79	2,848 (19.7)	2,877 (19.7)
≥80	1,635 (11.3)	1,702 (11.6)
Family structure		
Living alone	636 (4.4)	1,981 (13.6)
Living with family	12,338 (85.3)	11,120 (76.2)
Others	1,308 (9.0)	1,257 (8.6)
Unknown	183 (1.3)	242 (1.7)
Marital status		
Married	13,049 (90.2)	9,369 (64.2)
Widow	919 (6.4)	4,262 (29.2)
Divorced	221 (1.5)	466 (3.2)
Never married	113 (0.8)	288 (2.0)
Unknown	163 (1.1)	215 (1.5)
Household income (million yen/year)		
<2.00	5,281 (36.5)	5,587 (38.3)
2.00–3.99	6,284 (43.4)	5,137 (35.2)
≥4.00	1,864 (12.9)	1,634 (11.2)
Unknown	1,036 (7.2)	2,242 (15.3)
Current employment		
Yes	4,562 (31.5)	2,661 (18.2)
No	8,653 (59.8)	7,608 (52.1)
Never	438 (3.0)	2,417 (16.6)
Unknown	812 (5.6)	1,914 (13.1)
Years of schooling		
≤9	5,386 (37.2)	6,720 (46.0)
10–12	5,182 (35.8)	5,594 (38.3)
≥13	3,726 (25.8)	2,045 (14.0)
Unknown	171 (1.2)	241 (1.7)
Comorbidity		
No	6,181 (42.7)	8,044 (55.1)
Yes	4,227 (29.2)	2,645 (18.1)
Unknown	4,057 (28.0)	3,911 (26.8)

Data are numbers (percentages).

During the 3-year follow-up period, the number of participants with incident depressive symptoms was 2,383 (16.5%) in men and 2,298 (15.7%) in women (GDS-15 at the 2013 wave: median 2; interquartile range, 1–4 in both men and women). The null model of multilevel logistic regression model (Table 3) showed that the likelihood of developing depressive symptoms varied across school districts (random-effects variance 0.031; standard error, 0.014, MOR 1.23 in men; random-effects variance 0.041; standard error, 0.017 and MOR 1.27 in women; refer to the footnote). Community-level civic participation was inversely associated with the development of depressive symptoms among both sexes (AOR per one SD increase, 0.93; 95% CI, 0.88–0.99 in men; AOR 0.94; 95% CI, 0.88–0.997 in women; Table 3). Conversely, community-level social cohesion and reciprocity were not associated with the onset of depressive symptoms. Individual-level responses to civic participation, social cohesion, and reciprocity were all inversely significantly associated with the onset of depressive symptoms in both sexes (model 1). The AOR of depressive symptoms for individual responses to the civic participation scale was 0.75 (95% CI, 0.71–0.80) among men and 0.82 (95% CI, 0.78–0.87) among women, that for social cohesion was 0.77 (95% CI, 0.74–0.81) among men and 0.78 (95% CI, 0.75–0.82) among women, and that for reciprocity was 0.88 (95% CI, 0.85–0.91) among men and 0.88 (95% CI, 0.83–0.92) among women.

**Table 3.** Adjusted odds ratios^a^ for the onset of depressive symptoms by social capital indicators: Multilevel logistic regression, Japan Gerontological Evaluation Study (2010–2013)

	Model 1: two levels of social capital modeled separately				Model 2: two levels of social capital modeled simultaneously			
	
	*n*	AOR (95% CI)	Var RE (SE)	MOR	*n*	AOR (95% CI)	Var RE (SE)	MOR
**Men**								
**Civic participation**					12,051		0.014 (0.013)	1.15
Community level	14,465	0.93 (0.88, 0.99)^*^	0.015 (0.011)	1.15		0.96 (0.90, 1.02)		
Individual level	12,051	0.75 (0.71, 0.80)^**^	0.015 (0.013)	1.15		0.76 (0.72, 0.80)^**^		
**Social cohesion**					13,828		0.021 (0.013)	1.18
Community level	14,465	1.01 (0.95, 1.07)	0.019 (0.012)	1.17		1.05 (0.99, 1.12)		
Individual level	13,828	0.77 (0.74, 0.81)^**^	0.021 (0.013)	1.19		0.77 (0.73, 0.80)^**^		
**Reciprocity**					13,717		0.015 (0.012)	1.15
Community level	14,465	0.95 (0.89, 1.01)	0.017 (0.011)	1.16		0.95 (0.89, 1.02)		
Individual level	13,717	0.88 (0.85, 0.91)^**^	0.016 (0.012)	1.16		0.88 (0.85, 0.91)^**^		
**Women**								
**Civic participation**					10,871		0.031 (0.019)	1.23
Community level	14,600	0.94 (0.88, 0.997)^*^	0.023 (0.014)	1.19		0.99 (0.92, 1.06)		
Individual level	10,871	0.82 (0.78, 0.87)^**^	0.031 (0.019)	1.23		0.82 (0.78, 0.87)^**^		
**Social cohesion**					13,873		0.023 (0.015)	1.19
Community level	14,600	0.99 (0.93, 1.06)	0.026 (0.014)	1.21		1.04 (0.97, 1.11)		
Individual level	13,873	0.78 (0.75, 0.82)^**^	0.024 (0.015)	1.20		0.78 (0.75, 0.81)^**^		
**Reciprocity**					13,720		0.027 (0.015)	1.21
Community level	14,600	1.01 (0.95, 1.08)	0.026 (0.015)	1.21		1.01 (0.95, 1.08)		
Individual level	13,720	0.88 (0.83, 0.92)^**^	0.027 (0.015)	1.21		0.87 (0.83, 0.92)^**^		

AOR, adjusted odds ratio; CI, confidence interval; MOR, median odds ratio; SE, standard error; Var RE, random-effects variance.

MOR: $\text{exp}(\sqrt{2\times \text{Var}\;\text{RE}}\times 0.6745)$.

^a^Per 1 standard deviation unit change in the social capital component score. In the null model, Var RE (SE) and MOR was 0.031 (0.014) and 1.23 for men, and 0.041 (0.017) and 1.27 for women, respectively. Covariates adjusted: age, family structure, marital status, household income, current employment, years of schooling, and comorbidity.

^*^P < .05, ^**^P < .001.

When we modeled both community-level social capital and individual responses to each component simultaneously, the point estimates in community-level scores were attenuated. Specifically, community-level civic participation increased from 0.93 to 0.96 in men and from 0.94 to 0.99 in women (model 2, Table 3). The AORs of the individual-level social capital components were similar those for model 2 and model 1.

During the maximum 7-year follow-up period, our three-wave data set had 817 men (21.6%) and 798 women (21.9%) experiencing the onset of depressive symptoms. Discrete-hazard analyses using the data set showed statistically less clear but similar associations (eTable 4, eTable 5, and eTable 6).

## DISCUSSION

This prospective longitudinal study found that community-level civic participation was inversely associated with the onset of depressive symptoms among older people in Japan. No such association was found in relation to community-level social cohesion and reciprocity. On the other hand, all individual-level responses to community-level social capital components were inversely associated with the onset of depressive symptoms; a one-SD increase in each of the social capital scores was linked to 12–24% lower odds for the onset of depressive symptoms. When we modeled community- and individual-level scores simultaneously, the statistical significance of the association between community-level civic participation and depressive symptoms onset was attenuated.

Moore et al simultaneously investigated the effects of individual- and neighborhood-level social cohesion on depression among adults aged 45–84 years in the United States,^18^ and they found that neither an increase in neighborhood-level nor in individual-level social cohesion was significantly associated with a change in depression. The inverse association between community-level social cohesion and depression reported by Mair et al was not adjusted for individual-level social cohesion.^39^ The discrepancy between the current findings and those of these recent studies might have resulted from measurement issues. Using the community-level social capital measures utilized in the present study, Saito et al reported that civic participation is more closely linked with positive health outcomes than with community-level social cohesion, probably because community-level social cohesion is measured more objectively, and that it might have less measurement bias that is caused by the absence of those who do not trust others in the community.^22^ An alternative explanation may the variations caused by the country in which the study was conducted.

The findings observed in this study in relation to individual group participation were consistent with those reported in previous studies.^40^^–^^42^ For example, an 18-year longitudinal survey in Taiwan conducted by Chiao et al revealed that social participation was associated with fewer depressive symptoms in an older population.^40^ Croezen et al reported that, among the population aged 50 years or older in 10 European countries, participation in religious organizations was associated with a decline in depressive symptoms, while participation in political/community organizations was linked with an increase in depressive symptoms, highlighting the differences in the direction of the association by the type of social activity.^41^ Park et al also reported different effects of the type of activity; participating in leisure activities was associated with fewer depressive symptoms, while participating in volunteer activities was not.^42^

The non-significant association between community-level social capital and depressive symptoms after adjusting for individual-level social capital suggests that individual participation mediated the association between community-level social capital and the onset of depressive symptoms. This finding is in line with a recent intervention that reported that a municipality project on strategically building community social gathering opportunities increased individuals’ group participation, resulting in better health outcomes.^3^^,^^43^

Although the present study did not suggest the contextual effects of community-level social cohesion and reciprocity on depressive symptoms, individual responses to them were strongly associated with lower risks for depressive symptoms. Other recent studies have also reported that individual-level social cohesion and reciprocity are associated with fewer depressive symptoms in the older population^42^^,^^44^ and the general population.^42^^,^^44^^–^^46^ For example, Park et al conducted a longitudinal study (2006–2013) among older women in South Korea and reported that interpersonal trust and reciprocity were both associated with fewer depressive symptoms.^42^ In their cross-sectional study, Forsman et al reported that trust in friends and neighbors was associated with less depression among older adults in Sweden and Finland.^44^ In their prospective study conducted among the United States’ general population, Fujiwara and Kawachi showed that trusting neighbors was inversely associated with the risk of major depression.^45^ In 2007, Kim et al showed that interpersonal trust in 2006 was inversely associated with developing depression among the general population in South Korea,^46^ while the significant inverse association observed in relation to reciprocity was attenuated and became non-significant when only healthy people were included in the analysis.

There are some limitations to the present study. First, this study was a postal survey and might have been difficult for those with severe depression or other serious health problems to complete. It is possible that our participants were healthier than the general population and they might not represent the older population in Japan adequately. Second, a selection bias might also exist due to attrition: those with lower social capital may have been more likely to drop out in the follow-up surveys. Third, our social capital score lacked a few questions from the original social capital scale used by Saito et al.^22^ However, our factor analysis identified the same three components as those in their study, suggesting that our measure properly measured the components of community social capital adequately. Fourth, as social capital and depressive symptoms were based on a questionnaire, these responses are subject to common method biases, and those who were depressed might have perceived themselves as having lower social capital irrespective of their actual social capital. Finally, when we created community-level social capital variables, we depended solely on individual responses collected from older individual who participated in the survey. While Saito et al^22^ confirmed the validity of the scores, excluding younger generations and those who did not participate in the survey when calculating community-level social capital might have resulted in measurement error.

Despite these limitations, this study has extended the findings of previous studies on the association between social capital and depressive symptoms in many ways. First, our longitudinal study investigated the association among larger numbers of participants and community units as compared to two previous reports,^18^^,^^39^ while a still larger number of community units might have been necessary to examine the effect of community-level social capital. Second, our finding was robust in that it was also confirmed by the analysis using three-wave data obtained from 25 school districts. Third, we used study-specific social capital scores, which were previously shown to be associated with self-rated health and depression in Japan.

This study suggests that the promotion of civic participation might help mitigate the social impact of late-life depressive symptoms. While more research is needed, community programs for older adults, which focus on creating more opportunities for group participation, might increase actual individual participation, resulting in better health.^43^

## APPENDIX A. SUPPLEMENTARY DATA

eTable 1. Adjusted odds ratios^a^ for the onset of depressive symptoms by individual- and community-level social capital among total population: Multilevel logistic regression, Japan Gerontological Evaluation Study (2010–2013)

eTable 2. Factor loadings of the individual- and community-level social capital scores without participation in “study or cultural groups” and “activities for teaching specific skills” in civic participation in the Japan Gerontological Evaluation Study (2010–2013)

eTable 3. Factor loadings of the individual-level^a^ social capital scores without participation in “study or cultural groups” and “activities for teaching specific skills” in civic participation, and “community attachment” in social cohesion, Japan Gerontological Evaluation Study (2006–2010–2013)

eTable 4. Individual- and community-level distributions of civic participation, social cohesion, and reciprocity scores at baseline (2006 or 2010), Japan Gerontological Evaluation Study (2006–2010–2013)

eTable 5. Characteristics of the study participants at baseline (2006 or 2010) in the Japan Gerontological Evaluation Study (2006–2010–2013)

eTable 6. Adjusted odds ratios^a^ for the onset of depressive symptoms by individual- and community-level social capital: Multilevel logistic regression, Japan Gerontological Evaluation Study (2006–2010–2013)
