# Supplementary material for: Community Social Capital and Depressive Symptoms Among Older People in Japan: A Multilevel Longitudinal Study
Source: J Epidemiol. 2019 Oct 5;29(10):363–9. doi: 10.2188/jea.JE20180078 (PMC6737188; doi:10.2188/jea.JE20180078)
Supplement: Supplementary file 1 [file je-29-363-s001.pdf]

**eTable 1.** Adjusted odds ratios[a] for the onset of depressive symptoms by individual- and community-level social capital among total population: Multilevel logistic regression, Japan Gerontological Evaluation Study (2010–2013)

| | Model 1: two levels of social capital modeled separately | | | | | Model 2: two levels of social capital modeled simultaneously | | | |
|---|---|---|---|---|---|---|---|---|---|
| | **n** | **AOR (95% CI)** | **Var RE (SE)** | **MOR** | ***p* for interaction** | **n** | **AOR (95% CI)** | **Var RE (SE)** | **MOR** |
| **Civic participation** | | | | | | 22,922 | | 0.012 (0.007) | 1.14 |
| Community level | 29,065 | 0.93 (0.90, 0.97) * | 0.011 (0.006) | 1.13 | 0.194 | | 0.97 (0.93, 1.02) | | |
| Individual level | 22,922 | 0.79 (0.75, 0.82) ** | 0.012 (0.007) | 1.14 | 0.034 | | 0.79 (0.76, 0.82) ** | | |
| **Social cohesion** | | | | | | 27,701 | | 0.015 (0.007) | 1.15 |
| Community level | 29,065 | 1.00 (0.96, 1.05) | 0.013 (0.006) | 1.14 | 0.816 | | 1.05 (1.00, 1.09) | | |
| Individual level | 27,701 | 0.78 (0.75, 0.80) ** | 0.016 (0.007) | 1.16 | 0.617 | | 0.77 (0.75, 0.80) ** | | |
| **Reciprocity** | | | | | | 27,437 | | 0.014 (0.007) | 1.15 |
| Community level | 29,065 | 0.98 (0.94, 1.03) | 0.013 (0.006) | 1.14 | 0.096 | | 0.98 (0.94, 1.03) | | |
| Individual level | 27,437 | 0.88 (0.85, 0.90) ** | 0.014 (0.07) | 1.15 | 0.922 | | 0.88 (0.85, 0.90) ** | | |

AOR, adjusted odds ratio; CI, confidence interval; MOR, median odds ratio; SE, standard error; Var RE, random-effects variance.

MOR: $(\exp\sqrt{2 \times \text{Var RE}} \times 0.6745)$.

[a]Per 1 standard deviation unit change in the social capital component score. In the null model, Var RE (SE) and MOR was 0.025 (0.008) and 1.20.

Covariates adjusted: sex, age, family structure, marital status, household income, current employment, years of schooling, and comorbidity.

*p* for interaction, interaction between sex and social capital.

$^{*}p < .05$, $^{**}p < .001$

**eTable 2.** Factor loadings of the individual- and community-level social capital scores without participation in "study or cultural groups" and "activities for teaching specific skills" in civic participation in the Japan Gerontological Evaluation Study (2010–2013)

| | Individual-level (n=44,702) | | | Community-level (n=295) | | |
|---|---|---|---|---|---|---|
| | Civic participation (F1) | Social cohesion (F2) | Reciprocity (F3) | Civic Participation (F1) | Social cohesion (F2) | Reciprocity (F3) |
| Volunteer group | 0.405 | - | - | 0.432 | - | - |
| Sports group | 0.610 | - | - | 0.857 | - | - |
| Hobby activity | 0.681 | - | - | 0.788 | - | - |
| Community trust | - | 0.782 | - | - | 0.933 | - |
| Norms of reciprocity | - | 0.677 | - | - | 0.797 | - |
| Community attachment | - | 0.549 | - | - | 0.732 | - |
| Received emotional support | - | - | 0.807 | - | - | 0.822 |
| Provided emotional support | - | - | 0.707 | - | - | 0.751 |
| Received instrumental support | - | - | 0.352 | - | - | 0.576 |
| Correlation coefficient | | | | | | |
| F1 & F2 | 0.197 (*P*=.000) | | | 0.264 (*P*=.000) | | |
| F2 & F3 | 0.159 (*P*=.000) | | | 0.173 (*P*=.002) | | |
| F1 & F3 | 0.244 (*P*=.000) | | | 0.460 (*P*=.000) | | |

Model fit scores of the confirmatory factor analysis were as follows:
Individual level: Chi-square(df) = 931.0(24), *p*-value < .001, Root Mean Square Error of Approximation (RMSEA) = 0.029, Comparative Fit Index (CFI) = 0.986, Tucker–Lewis index (TLI) = 0.979, Standardized Root Mean Square Residual (SRMR) = 0.022
Community level: Chi-square(df) = 92.0(24), *p*-value < .001, RMSEA = .098, CFI = 0.933, TLI = 0.899, SRMR = 0.063

**eTable 3.** Factor loadings of the individual-level[a] social capital scores without participation in "study or cultural groups" and "activities for teaching specific skills" in civic participation, and "community attachment" in social cohesion, Japan Gerontological Evaluation Study (2006–2010–2013)

| | **Individual-level (n=44,919)** | | |
|---|---|---|---|
| | **Civic participation (F1)** | **Social cohesion (F2)** | **Reciprocity (F3)** |
| Volunteer group | 0.404 | - | - |
| Sports group | 0.609 | - | - |
| Hobby activity | 0.681 | - | - |
| Community trust | - | 0.808 | - |
| Norms of reciprocity | - | 0.661 | - |
| Community attachment | - | - | - |
| Received emotional support | - | - | 0.810 |
| Provided emotional support | - | - | 0.704 |
| Received instrumental support | - | - | 0.351 |
| Correlation coefficient | | | |
| F1 & F2 | 0.185 (*P*=.000) | | |
| F2 & F3 | 0.159 (*P*=.000) | | |
| F1 & F3 | 0.219 (*P*=.000) | | |

Model fit scores of the confirmatory factor analysis were as follows:

Individual level: Chi-square(df) = 495.3 (17), *p*-value < .001, Root Mean Square Error of Approximation (RMSEA) = 0.025, Comparative Fit Index (CFI) = 0.991, Tucker–Lewis index (TLI) = 0.985, Standardized Root Mean Square Residual (SRMR) = 0.017

[a]We did not run a factor analysis for community-level social capital assessed based on the questions used in our three-wave analysis due to the limited sample size.

**eTable 4.** Individual- and community-level distributions of civic participation, social cohesion, and reciprocity scores at baseline (2006 or 2010), Japan Gerontological Evaluation Study (2006–2010–2013)

| | Individual level[a] | | | | | | Community level[c] | |
|---|---|---|---|---|---|---|---|---|
| | | | 0 (no score) | 1 | 2 | 3 | | |
| | n | Median[b] | n (%) | n (%) | n (%) | n (%) | n | Median (IQR) |
| **Men** | | | | | | | | |
| Civic participation | 3,293 | -0.72 | 1,982 (60.2) | 743 (22.6) | 460 (14.0) | 108 (3.3) | 3,779 | 0.44 (0.01, 0.94) |
| Social cohesion | 3,645 | 0.78 | 707 (19.4) | 927 (25.4) | 2,011 (55.2) | - | 3,779 | -0.29 (-0.77, 0.51) |
| Reciprocity | 3,612 | 0.28 | 111 (3.1) | 86 (2.4) | 132 (3.7) | 3,283 (90.9) | 3,779 | -0.18 (-0.92, 0.65) |
| **Women** | | | | | | | | |
| Civic participation | 2,835 | -0.72 | 1,463 (51.6) | 706 (24.9) | 532 (18.8) | 134 (4.7) | 3,645 | 0.44 (-0.16, 0.94) |
| Social cohesion | 3,459 | 0.78 | 837 (24.2) | 868 (25.1) | 1,754 (50.7) | - | 3,645 | -0.22 (-0.77, 0.54) |
| Reciprocity | 3,383 | 0.28 | 54 (1.6) | 66 (2.0) | 58 (1.7) | 3,206 (94.8) | 3,645 | -0.12 (-0.78, 0.65) |

IQR, interquartile range.

[a]The social capital score at the individual level was the total number of "strongly or moderately agree/yes" responses in civic participation, social cohesion, and reciprocity, respectively (e.g., a 0 score indicated that none of items apply, while a 3 score indicated that all the items apply)

[b]Median of standardized social capital score.

[c]Standardized % of average "strongly or moderately agree/yes" responses in a school district.

**eTable 5.** Characteristics of the study participants at baseline (2006 or 2010) in the Japan Gerontological Evaluation Study (2006–2010–2013)

| | Men | Women |
|---|---|---|
| Age, years | | |
| 65–69 | 2,159 (57.1) | 1,921 (52.7) |
| 70–74 | 883 (23.4) | 911 (25.0) |
| 75–79 | 489 (12.9) | 509 (14.0) |
| ≥80 | 248 (6.5) | 304 (8.3) |
| Family structure | | |
| Living alone | 120 (3.2) | 387 (10.6) |
| Living with family | 3,210 (84.9) | 2,838 (77.9) |
| Others | 426 (11.3) | 385 (10.6) |
| Unknown | 23 (0.6) | 35 (1.0) |
| Marital status | | |
| Married | 3,451 (91.3) | 2,411 (66.2) |
| Widow | 212 (5.6) | 968 (26.6) |
| Divorced | 40 (1.1) | 101 (2.8) |
| Unmarried | 19 (0.5) | 69 (1.9) |
| Unknown | 57 (1.5) | 96 (2.6) |
| Household income (million yen/year) | | |
| <2.00 | 1,363 (36.1) | 1,273 (34.9) |
| 2.00–3.99 | 1,596 (42.2) | 1,214 (33.3) |
| ≥4.00 | 424 (11.2) | 369 (10.1) |
| Unknown | 396 (10.5) | 789 (21.7) |
| Current employment | | |
| Working | 1,432 (37.9) | 799 (21.9) |
| Not working | 1,983 (52.5) | 1,645 (45.1) |
| Never working | 152 (4.0) | 622 (17.1) |
| Unknown | 212 (5.6) | 579 (15.9) |
| Years of schooling | | |
| ≤9 | 930 (24.6) | 1,062 (29.1) |
| 10–12 | 667 (17.7) | 600 (16.5) |
| ≥13 | 327 (8.7) | 157 (4.3) |
| Unknown | 1,855 (49.1) | 1,826 (50.1) |
| Comorbidity | | |
| No | 2,424 (64.1) | 2,587 (71.0) |
| Yes | 815 (21.6) | 575 (15.8) |
| Unknown | 540 (14.3) | 483 (13.3) |

Data are numbers (percentages)

**eTable 6.** Adjusted odds ratios[a] for the onset of depressive symptoms by individual- and community-level social capital: Multilevel logistic regression, Japan Gerontological Evaluation Study (2006–2010–2013)

| | **Model 1: two levels of social capital modeled separately** | | | | **Model 2: two levels of social capital modeled simultaneously** | | | |
|---|---|---|---|---|---|---|---|---|
| | **n** | **AOR (95% CI)** | **Var RE (SE)** | **MOR** | **n** | **AOR (95% CI)** | **Var RE (SE)** | **MOR** |
| **Men** | | | | | | | | |
| **Civic participation** | | | | | 4,322 | | 0.0004 (0.012) | 1.02 |
| Community level | 5,060 | 0.97 (0.86, 1.09) | 0.012 (0.015) | 1.14 | | 1.02 (0.91, 1.14) | | |
| Individual level | 4,322 | 0.77 (0.70, 0.85) ** | 0.010 (0.012) | 1.04 | | 0.77 (0.70, 0.85) ** | | |
| **Social cohesion** | | | | | 4,837 | | 9.1e-33 (3.4e-17) | 1.00 |
| Community level | 5,060 | 0.94 (0.85, 1.04) | 0.006 (0.013) | 1.09 | | 0.96 (0.87, 1.05) | | |
| Individual level | 4,873 | 0.73 (0.67, 0.78) ** | 1.9e-33 (9.6e-18) | 1.00 | | 0.73 (0.67, 0.79) ** | | |
| **Reciprocity** | | | | | 4,823 | | 0.009 (0.014) | 1.12 |
| Community level | 5,060 | 1.00 (0.91, 1.09) | 0.012 (0.014) | 1.13 | | 0.99 (0.90, 1.08) | | |
| Individual level | 4,823 | 0.85 (0.79, 0.91) ** | 0.008 (0.014) | 1.11 | | 0.85 (0.80, 0.91) ** | | |
| **Women** | | | | | | | | |
| **Civic participation** | | | | | 3,699 | | 0.029 (0.023) | 1.22 |
| Community level | 4,883 | 0.93 (0.83, 1.05) | 0.029 (0.020) | 1.22 | | 1.00 (0.88, 1.14) | | |
| Individual level | 3,699 | 0.80 (0.73, 0.88) ** | 0.029 (0.023) | 1.22 | | 0.80 (0.73, 0.88) ** | | |
| **Social cohesion** | | | | | 4,641 | | 0.021 (0.017) | 1.18 |
| Community level | 4,883 | 0.96 (0.86, 1.08) | 0.028 (0.020) | 1.22 | | 0.98 (0.88, 1.10) | | |
| Individual level | 4,641 | 0.82 (0.76, 0.88) ** | 0.020 (0.017) | 1.18 | | 0.82 (0.76, 0.89) ** | | |
| **Reciprocity** | | | | | 4,533 | | 0.020 (0.019) | 1.18 |
| Community level | 4,883 | 0.98 (0.88, 1.09) | 0.030 (0.022) | 1.22 | | 1.00 (0.90, 1.11) | | |
| Individual level | 4,533 | 0.85 (0.78, 0.92) ** | 0.020 (0.017) | 1.18 | | 0.85 (0.78, 0.92) ** | | |

AOR, adjusted odds ratio; CI, confidence interval; MOR, median odds ratio; SE, standard error; Var RE, random-effects variance.

MOR: $\exp(\sqrt{2 \times \text{Var RE}} \times 0.6745)$.

[a]Per 1 standard deviation unit change in the social capital component score. In the null model, Var RE (SE) and MOR was 0.010 (0.014) and 1.12 for men, and 0.025 (0.019) and 1.20 for women, respectively. Covariates adjusted: age, family structure, marital status, household income, current employment, years of schooling, comorbidity, and years (2010 and 2013).

$^{*}p < .05$, $^{**}p < .001$
